# Long-Term Functional Outcome and Quality of Life in Long-Term Traumatic Brain Injury Survivors

**DOI:** 10.1089/neur.2023.0064

**Published:** 2023-11-22

**Authors:** Wivi Taalas, Rahul Raj, Juha Öhman, Jari Siironen

**Affiliations:** ^1^ Department of Neurosurgery, Helsinki University Hospital and University of Helsinki, Bridge Hospital, Helsinki, Finland.; ^2^ Department of Neurosurgery, Tampere University Hospital, Tampere, Finland.

**Keywords:** functional outcome, outcome, prognosis, quality of life, traumatic brain injury

## Abstract

Early functional outcome assessments of traumatic brain injury (TBI) survivors may underestimate the long-term consequences of TBI. We assessed long-term temporal changes in functional outcome and quality of life in intensive care unit-managed long-term TBI survivors. This prospective, longitudinal study included 180 patients admitted to a single university hospital during 2000–2002 alive at 15 years post-TBI. Baseline characteristics, including imaging information, were collected. Functional outcome was assessed early (6–24 months) and late (15 years) using the Glasgow Outcome Scale (GOS) and the extended GOS (GOSE). Quality of life was measured at 15 years using the EuroQol Five Dimensions Five Levels (EQ-5D-5L) questionnaire. GOS and GOSE were dichotomized into favorable and unfavorable outcome. An index score was computed for EQ-5D-5L results at 15 years by a standardized valuation protocol. Of 180 patients, 118 replied to 15-year questionnaires. Median age at time of injury was 34 years (interquartile range, 19–45). Using the GCS to assess TBI severity, 67% had a moderate-to-severe TBI. Ninety-seven percent had favorable early functional outcome, and 72% had late favorable functional outcome. Logistic regression found higher age, lower GCS, and Marshall CT III to significantly predict late unfavorable functional outcome. Higher age and Marshall CT III were significant predictors of functional outcome deterioration. Median EQ-5D-5L index score for all patients was 0.88 (0.66–1.00) and correlated positively with GOSE. Most long-term TBI survivors with early favorable outcome also have late favorable functional outcome. Higher age and diffuse brain injury are associated with neurological deterioration. Quality of life was strongly linked to functional outcome.

## Introduction

Traumatic brain injury (TBI) survivors face significant post-TBI physical, social, and emotional impairments^1,2^ that influence daily life and psychological well-being.^3^ These impairments occur after severe, moderate^4^ and mild TBIs.^5^ Most research has focused on outcome measures assessed 6–24 months post-TBI.^6–9^

However, post-TBI cognitive improvements continue for at least 5 years.^10^ Some studies have found notable amounts of patients to improve 2–20 years post-injury.^11–13^ Furthermore, up to 96% of TBI survivors have favorable outcomes at 20 years post-injury.^14^ Thus, studies assessing outcomes too early do not fully describe recovery, leading to type II errors. Other evidence suggests the outcomes of TBI patients to deteriorate over time. No consensus exists regarding the recovery trajectories of TBI patients 20 years post-TBI.^15^

Two main methods have been used to assess long-term TBI outcomes. The first method focuses on assessing temporal changes in outcomes by measuring the Glasgow Outcome Scale (GOS) or extended GOS (GOSE)^16,17^ at different time points.^12–14,18–20^ Previous results have varied, with some studies suggesting deteriorations in outcomes in the long term,^2,18^ improvements over time,^12^ or early outcomes to persist throughout later years.^13,20^

The second method refers to outcome prediction, which focuses on predicting outcomes at a distinct time point with admission characteristics as outcome predictors.^7,8,21–24^ Younger age^18–21^ and higher Glasgow Coma Scale (GCS)^12,14^ are linked to favorable long-term post-TBI outcomes. The influence of other factors on long-term post-TBI outcomes remains unclear. Measures of injury severity, including pupillary light reactivity^7,8,22,23^ and Marshall computed tomography (CT),^24,25^ have been found to be predictive of shorter-term functional outcomes.

Few studies to date have assessed the relationship between functional outcomes and life quality. One recent study reported a strong positive correlation between the patient EuroQol Five Dimensions Five Levels (EQ-5D-5L) index^26^ and GOS scores.^27^

This study aimed to 1) examine changes between early (6–24 months) and late functional outcome (15 years), 2) assess how patient admission characteristics predict functional outcome and risk for functional outcome deterioration, and 3) investigate the relationship between functional outcome and quality of life in long-term TBI survivors.

We hypothesized functional outcome to deteriorate between early and late assessments^2,18^; younger age,^18–21^ higher GCS,^12,14^ reactive pupils,^7,8,22,23^ lower Marshall CT class,^24^ and male sex^18^ to predict favorable functional outcome; and better functional outcome to be associated with higher life quality.^27^

## Methods

### Study setting and patient population

This prospective, longitudinal panel study was conducted using the consecutive data of intensive care unit (ICU) TBI patients managed at Helsinki University Hospital during April 10, 2000 to November 21, 2002. All patients alive at 15 years post-injury fitting the inclusion criteria were asked to participate. We excluded patients with injuries older than 24 h, those whose primary neurosurgical care was given elsewhere, foreigners, and those with missing admission data. Patients <16 years were not excluded. Patients were contacted by letter or over the phone. The study was approved by the ethics committee at Helsinki University Hospital (45/2006, Dro 239/E9/06). The STROBE (Strengthening the Reporting of Observational Studies in Epidemiology) guidelines for observational studies were followed.^28^

### Definition of variables

Age was considered at the time of TBI. GCS score and pupillary light reactivity were measured upon admission by the treating neurosurgeon and recorded. We defined severe TBI as GCS 3–8, moderate TBI as GCS 9–12, and mild TBI as GCS 13–15. We classified admission head CT scans according to the Marshall CT classification.^25^ We combined Marshall CT classes V (evacuated mass lesion) and VI (non-evacuated mass lesion) into one class (V).

#### Functional outcome

We assessed early functional outcome at 6–24 months post-injury (GOS, October 2000 to June 2002) because varying numbers of patients replied to surveys at 6, 12, and 24 months. Late functional outcome was considered at 15 years post-injury (GOSE, January 2018 to July 2018). For early functional outcome, GOS 1–3 was considered unfavorable and 4–5 favorable. For late functional outcome, 1–4 was considered unfavorable and 5–8 favorable. GOS questionnaires (early functional outcome) were assessed by letters sent to the patient or next-of-kin. GOSE questionnaires (late functional outcome) were assessed by letters sent to the patient or next-of-kin. If the patient or next-of-kin was not reached by mail, all contact attempts were made by telephone. For patients with multiple GOS assessments (within 6–24 months), we considered the time point furthest away from the TBI, given that it is better reflective of the early post-injury recovery.^10^

#### Quality of life

We measured quality of life using the EQ-5D-5L at 15 years post-injury. EQ-5D-5L is a health-related quality of life measure introduced by the EuroQol group in 2009.^26^ The EQ-5D-5L questionnaire was sent along with the 15-year GOSE survey between January 2018 to July 2018 by letter. If the patient or next-of-kin was not reached by mail, all contact attempts were made by telephone. EQ-5D-5L results were computed at 15 years, with each parameter (mobility, self-care, usual activities, pain and discomfort, anxiety, and depression) measured from 1 to 5 (ranging from no to extreme problems) and self-rated health measured on a visual scale (Visual Analog Scale; VAS) from 0 to 100.

The EQ-5D-5L was functionalized by converting EQ-5D-5L values to an index score where negative numbers indicate health states worse than death, 0 a health state comparable to death, and 1 a state of perfect health.

Owing to the fact that a Finnish standardized valuation protocol (EuroQol Valuation Technology) based on the recommended composite time trade-off and/or discrete choice experiment for the EQ-5D-5L has not been developed, index scores were derived using the Danish value set.^29^ Given that Denmark is a Nordic country, the population-based EQ-5D-5L index values can be considered similar to those of the Finnish population.

### Statistical analyses

We used IBM SPSS (version 29.0; IBM Corp., Armonk, NY) and R Studio (version 2021.09.1; R Foundation for Statistical Computing, Vienna, Austria) for the statistical analyses.

We presented categorical variables as numbers and percentages. We assessed continuous data for normality. All continuous data were skewed and are presented as medians with interquartile ranges (IQRs).

We created two separate binary logistic regression analyses to assess the association between patient admission characteristics (age, sex, GCS score, pupillary light reactivity, and Marshall CT) and late functional outcome (GOSE 1–4 unfavorable outcome, GOSE 5–8 favorable outcome), as well as functional outcome deterioration (early GOS 4–5 to late GOSE 1–4). Patients with one missing value for any predictor were excluded from regression analyses. We used Box-Tidwell tests to ensure the linearity of log odds of the continuous variables (age, GCS) with binary outcome. We examined tolerance and variance inflation factor (VIF) to avoid multi-collinearity between predictors.

We assessed the relationship between EQ-5D-5L index scores and functional outcome graphically by a scatter plot of mean values with confidence intervals (CIs). We used a Mann-Whitney U test to assess the differences between index scores for patients whose functional outcomes remained stable and improved compared to those whose functional outcomes deteriorated. We used a Levene's test for equality of variances to determine the homogeneity of variances of the two groups assessed in the Mann-Whitney U test. Patients with one missing value for any EQ-5D-5L dimension were excluded from index score analyses.

## Results

### Baseline characteristics

**FIG. 1.**
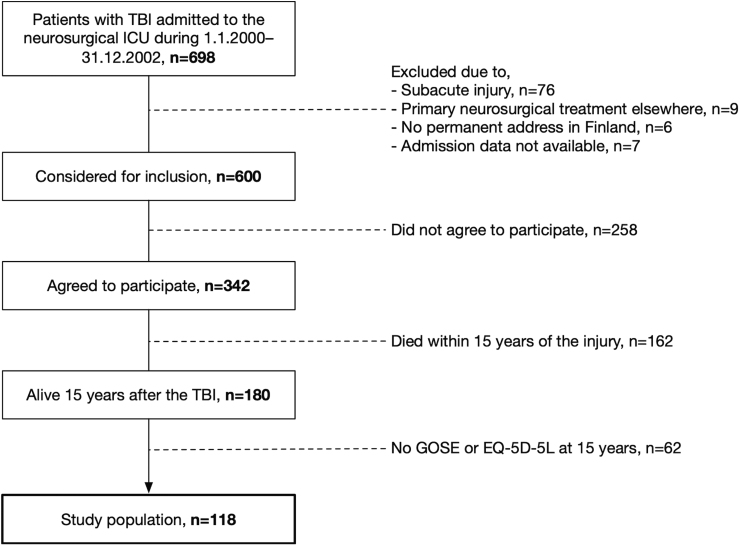
Flowchart demonstrating patients replying to functional outcome and quality-of-life surveys. ICU, intensive care unit; GOSE, Glasgow Outcome Scale-Extended; TBI, traumatic brain injury.

Of 698 patients admitted to the neurosurgical ICU from January 2000 to the end of December 2002, 600 fit the inclusion criteria and 342 patients agreed to participate (Fig. 1). Of these, 180 were alive at the time of 15-year post-TBI follow-up (assessed in July 2017), and 118 replied to follow-up questionnaires. Differences in baseline characteristics between patients who replied and did not reply to follow-up questionnaires are shown in Supplementary Table S1. Patients who did not reply at 15 years post-TBI were older (median, 41 vs. 34 years), had similar GCS scores (66% vs. 67% moderate-to-severe TBI), and had similar normal pupillary responsiveness (80% vs. 79%), compared to those who replied. Patients who died before the 15-year follow-up were older (median, 55 vs. 34 years), had similar GCS scores (68% vs. 67% moderate-severe TBI), and had similar normal pupillary responsiveness (72% vs. 79%), compared to those who did not die and were eligible for participation (Supplementary Table S2). The Kaplan-Meier survival curve depicting cumulative survival within 15 years of injury demonstrates approximately a 0.1 drop within the first month post-TBI and a more gradual decrease thereafter (Supplementary Fig. S5).

Characteristics of patients with favorable and unfavorable functional outcomes at 15 years post-TBI are shown in Table 1. Patients with favorable functional outcomes were younger (median, 28 vs. 43 years), had higher GCS scores (33% vs. 18% mild TBI), more often had normal pupillary light responsiveness (81% vs. 73%), and milder CT findings (Marshall CT II class, 67% vs. 40%) than patients with unfavorable outcome 15 years post-injury.

**Table 1.** Differences in Characteristics Between Patients With Favorable and Unfavorable Outcomes at 15 Years Post-TBI

Variable	All patients (*N* = 118)	Unfavorable outcome GOSE 1–4 (*N* = 33)	Favorable outcome GOSE 5–8 (*N* = 85)
Age of admission, median (IQR)	34 (19–45)	43 (33–54)	28 (15–42)
Sex (%)			
Male	88 (75)	27 (82)	61 (72)
Female	30 (25)	6 (18)	24 (28)
GCS score (%)			
3–8	50 (42)	20 (61)	30 (35)
9–12	29 (25)	7 (21)	22 (26)
13–15	34 (29)	6 (18)	28 (33)
NA	5 (4)	0 (0)	5 (6)
Pupil responsiveness (%)			
Bilaterally unresponsive	11 (9)	4 (12)	7 (8)
Unilaterally unresponsive	11(9)	5 (15)	6 (7)
Responsive	93 (79)	24 (73)	69 (81)
NA	3 (3)	0 (0)	3 (4)
Marshall CT (%)			
I	0 (0)	0 (0)	0 (0)
II	70 (59)	13 (40)	57 (67)
III	15 (13)	7 (21)	8 (9)
IV	7 (6)	2 (6)	5 (6)
V	26 (22)	11 (33)	15 (18)
Cause of injury (%)			
Fall from ground level	33 (28)	13 (40)	20 (24)
Fall from height	13 (11)	1 (3)	12 (14)
Traffic accident	40 (34)	11 (33)	29 (34)
Interpersonal violence	10 (8)	2 (6)	8 (9)
Other	14 (12)	3 (9)	11 (13)
Unknown	8 (7)	3 (9)	5 (6)


All percentages rounded to the nearest whole number.

TBI, traumatic brain injury; IQR, interquartile range; GCS, Glasgow Coma Scale; NA, not accessible; CT, computed tomography; GOSE, Glasgow Outcome Scale-Extended.

### Temporal changes in functional outcomes

**FIG. 2.**
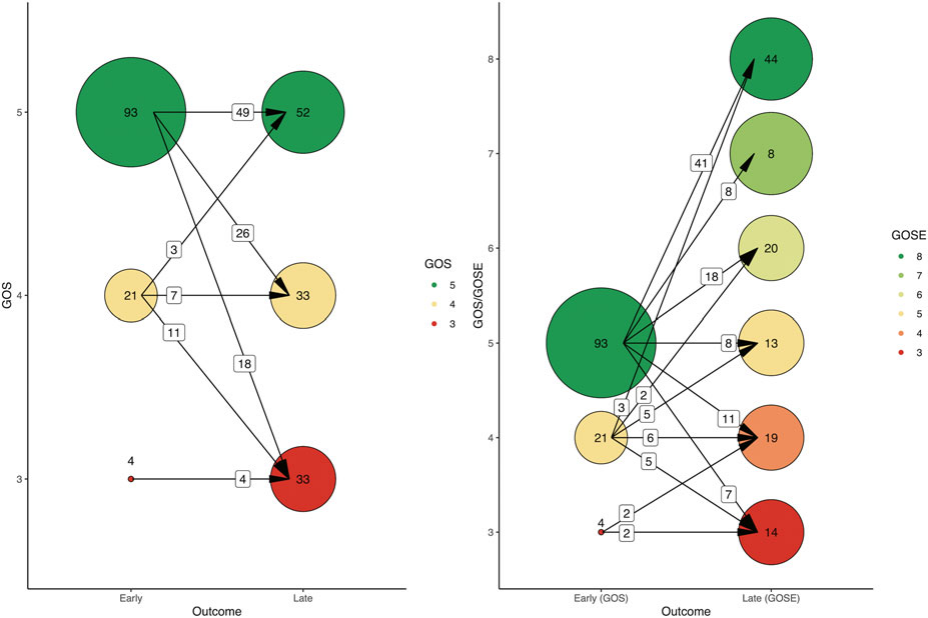
Distribution of outcomes at different time-points of measurement. Circles represent the number of patients in each outcome category. Arrows represent the number of patients moving between outcome categories. Data were collected as shown on the right. In the left figure, the good recovery, moderate disability, and severe disability brackets of the GOSE have been combined into corresponding GOS categories. GOS, Glasgow Outcome Scale; GOSE, Glasgow Outcome Scale-Extended.

Early outcomes were considered for 59 patients at 24 months, 48 patients at 12 months, and 11 patients at 6 months. Early patient functional outcomes were nearly completely favorable (97%). Patients with favorable early outcome were older (median, 34 vs. 30 years), more often female (26% vs. 0%), had higher GCS scores (30% compared to 0% mild TBI), more frequently normal pupillary light reactions (79% vs. 75%), and milder CT findings (Marshall CT III–V class, 40% vs. 50%) (Supplementary Table S3). Of the 97% of patients with early favorable functional outcomes, 72% retained favorable late functional outcome. The 3% of patients with early unfavorable outcomes retained unfavorable late functional outcomes on follow-up. Eighteen of 93 (19%) patients with early GOS 5 and 11 of 21 (52%) patients with an early GOS 4 deteriorated to unfavorable functional status on follow-up (Fig. 2).

Patients whose functional outcome deteriorated were older (41 vs. 27 years), had lower GCS scores (80% vs. 55% moderate-to-severe TBI), less frequently normal pupillary light reactions (73% vs. 84%), and more severe CT findings (Marshall CT III–V class, 53% vs. 30%) than patients with a stable/improved functional outcome (Table 2).

**Table 2.** Characteristics of Patients Whose Functional Outcome Remained the Same, Deteriorated, or Improved

Variable	Functional outcome improved or stayed the same (*N* = 63)	Functional outcome deteriorated (*N* = 55)
Age of admission, median (IQR)	27 (14.0–40.0)	41 (20.0–53.0)
Sex (%)		
Male	47 (75)	41 (75)
Female	16 (25)	14 (25)
GCS score (%)		
3–8	24 (38)	26 (47)
9–12	11 (17)	18 (33)
13–15	23 (37)	11 (20)
NA	5 (8)	0 (0)
Pupil responsiveness (%)		
Bilaterally unresponsive	5 (8)	6 (11)
Unilaterally unresponsive	4 (6)	7 (13)
Responsive	53 (84)	40 (73)
NA	1 (2)	2 (3)
Marshall CT (%)		
I	0 (0)	0 (0)
II	44 (70)	26 (47)
III	4 (6)	11 (20)
IV	5 (8)	2 (4)
V	10 (16)	16 (29)
Cause of injury (%)		
Fall from ground level	14 (22)	19 (35)
Fall from height	10 (16)	3 (6)
Traffic accident	20 (32)	20 (36)
Interpersonal violence	6 (9)	4 (7)
Other	10 (16)	4 (7)
Unknown	3 (5)	5 (9)


All percentages rounded to the nearest whole number.

IQR, interquartile range; GCS, Glasgow Coma Scale; NA, not accessible; CT, computed tomography.

### Binary logistic regression to predict long-term functional outcomes

Of the 118 patients, 8 were excluded from the multi-variable logistic regression analysis because of missing data (5 due to missing GCS, 3 due to missing pupillary reactivity).

Logistic regression analyses showed higher age, higher GCS, and Marshall CT III compared with II to significantly predict unfavorable functional outcome at 15 years post-injury. Higher age of admission and Marshall CT III were predictors of functional outcome deterioration (Table 3).

**Table 3.** Risk for Late Unfavorable Functional Outcome and Functional Outcome Deterioration

Variable	Risk for late unfavorable functional outcome (OR, 95% CI)	Risk for functional outcome deterioration (OR, 95% CI)
Age of admission^1^,^*^,^**^^2^	1.04 (1.01–1.08)	1.04 (1.01–1.07)
Sex, female as the reference category	2.23 (0.68–7.32)	0.82 (0.31–2.19)
GCS score^1^*^^	0.86 (0.75–0.98)	0.90 (0.80–1.02)
Pupil responsiveness, unresponsive (unilaterally + bilaterally) as the reference category		
Responsive	0.65 (0.19–2.25)	0.54 (0.16–1.86)
Marshall CT, II used as the reference category		
III^1,^*^,^**^2^	4.69 (1.21–18.16)	3.95 (1.02–15.34)
IV	0.56 (0.08–3.78)	0.23 (0.03–1.54)
V	1.95 (0.54–7.11)	1.54 (0.46–5.15)


Significant (*p* < 0.05) predictors for risk of unfavorable late functional outcome marked with^1^*^^ and for functional outcome deterioration marked with^2^**^^.

GCS, Glasgow Coma Scale; CT, computed tomography; OR, odds ratio; CI, confidence interval.

The Box-Tidwell test to ensure linearity of log odds of the continuous variable with the outcome was insignificant for all models (all *p* values, >0.1). For all models, tolerances were >0.1 and VIFs <10, indicating absence or little multi-collinearity.

### Relationship between EuroQol Five Dimensions Five Levels index scores and 15-year Glasgow Outcome Scale-Extended

**FIG. 3.**
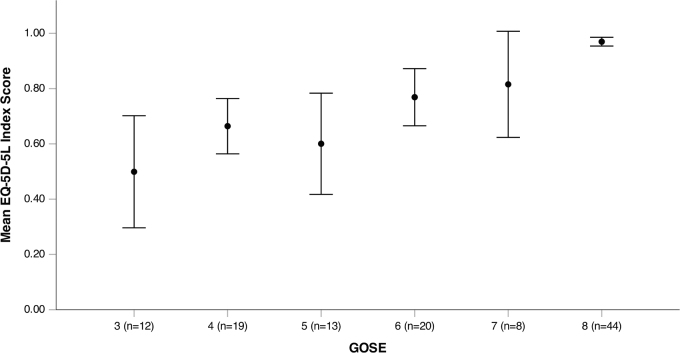
Relationship between EQ-5D-5L index scores and GOSE at 15 years. Error bars represent 95% confidence intervals (CI). Mean index scores generally increased along with the GOSE. GOSE 3 mean 0.50 (SD, 0.32), GOSE 4 mean 0.66 (SD, 0.21), GOSE 5 mean 0.60 (SD, 0.30), GOSE 6 mean 0.77 (SD, 0.22), GOSE 7 mean 0.82 (SD, 0.23), GOSE 8 mean 0.97 (SD, 0.52). EQ-5D-5L, EuroQol Five Dimensions Five Levels; GOSE, Glasgow Outcome Scale-Extended; SD, standard deviation.

Of the 118 patients, 2 were missing one dimension of the EQ-5D-5L and an index score could not be derived. The median index score for all patients was 0.88 (0.66–1.00). Except for those with a GOSE 5, mean index score increased with each increase in GOSE (Fig. 3). As shown in Supplementary Figure S1, 56% of variance in the EQ-5D-5L index scores was explained by the VAS, and there is a positive correlation between index scores and VAS.

### Linear regression to predict long-term quality of life

None of the factors included in the linear regression model showed any statistically significant association with long-term EQ-5D-5L index score (Supplementary Table S4).

### Differences in EuroQol Five Dimensions Five Levels index scores according to temporal change in functional outcomes

A Mann-Whitney U test comparing mean ranks demonstrated that if functional outcome remained stable or improved (*n* = 63), index score was significantly higher (mean rank = 76.93) compared to if functional outcome deteriorated (*n* = 53, mean rank = 36.59, *p* < 0.001; Supplementary Figs. S2–S4).

## Discussion

### Key findings

This prospective, longitudinal 15-year study found a large proportion of late favorable functional outcomes (72%) and quality of life (median 0.88 from a scale of negative numbers to a maximum of 1) among long-term TBI survivors, most of which had an early favorable functional outcome (97%). More than half (53%) of patients' functional outcomes stayed the same/improved between early and late measurements. Older age upon admission, lower GCS, and Marshall CT III versus II were significant predictors of 15-year unfavorable functional outcome. Older age on admission and Marshall CT III versus II significantly predicted risk for functional outcome deterioration. We found a positive relationship between GOSE and EQ-5D-5L index score at 15 years post-TBI. Quality of life was significantly higher (*p* < 0.001) if functional outcome remained the same/improved between early and late measurements.

### Comparison with previous studies

This study presents an optimistic view of functional outcome recovery at 15 years post-TBI in a sample of patients surviving in the long term with favorable functional outcomes at 6–24 months (97% favorable; 79% GOS 5 and 18% GOS 4). Previous research has found up to 92% of favorable outcomes at 2 years in moderate-severe TBI survivors.^18^ Our sample consisted of TBI patients alive at 15 years post-TBI, excluding those with the poorest prognosis dying within 15 years as shown by the initial rapid dropoff in the Kaplan-Meier curve (Supplementary Fig. S5). Given that more patients with early low GOS (1–3) die before long-term follow-up^20^ and decline participation,^33^ long-term studies spanning over 10 years may find more early favorable functional outcomes.^6,11^ This trend is not supported by all research. For example, in a study describing outcome trajectories of a severe TBI population from 1 to 10–15 years, the distribution between favorable and unfavorable outcomes at 1 year post-TBI was approximately equal.^20^ In the current sample, 22% of patients were <16 years and 29% had mild TBI, contributing to more favorable early outcomes than found previously.^12,18–21^

The finding of 72% favorable late functional outcomes is in line with previous research finding variable proportions (59–96%) of favorable outcomes at 10–20 years post-TBI.^14,19,20^ Previous research has found one quarter to one third of TBI patients to experience functional deteriorations around 10 years post-TBI, supporting the findings of our study.^1,2,20^ Less patients remained stable or improved between outcome measures (53%) compared to previous studies (63–69%).^2,18,20^ Patients with outcome deterioration were older compared to patients with stable/improved outcomes (median ages, 41 vs. 27 years). In the mild TBI group, survivors with unfavorable late functional outcome were older than those with late favorable outcome (median ages, 58 vs. 27). Mild TBI survivors experiencing outcome deterioration were also older than those with stable/improved outcomes (median ages, 47 vs. 28). These findings suggest age-related deterioration to contribute importantly to 15-year post-TBI outcomes.

We found age, GCS, and Marshall CT to predict 15-year outcomes and/or outcome deterioration. A wealth of evidence supports age as an important predictor of long-term post-TBI outcomes.^18–21^ We found age to significantly predict both long-term functional outcomes and outcome deterioration. Inclusion of patients not replying to outcome surveys with older median age would likely have increased unfavorable functional outcomes. Long-term functional outcome and functional outcome deterioration were also predicted by Marshall CT category III versus II. The absence of basal cisterns characteristic to Marshall CT III has been shown to predict 6-month outcomes.^7,34^ We propose this CT finding to predict longer-term functional outcomes by being an indirect marker of diffuse brain injury predisposing to neurodegeneration.^35^ Marshall CT IV was not associated with neurological deterioration, probably attributable to high mortality among these injuries.

GCS is predictive of both shorter-term,^7^ as well as longer-term functional outcomes,^12,14^ and was found to significantly predict late unfavorable outcomes. Eight percent of patients with initially absent pupillary light reactivity had favorable long-term favorable outcome, and 8% of those with initially absent pupillary reactions had stable/improved functional outcomes. Abnormal pupillary light reactivity is linked to unfavorable outcomes at up to 6 months post-TBI^7,8,22,23^ and is used to determine the withdrawal of care in the ICU.^36^ A subset of patients with initially absent pupillary light reactivity have favorable long-term outcomes. Men were also found to have better long-term outcomes, in line with some,^18,30^ but not all,^31^ previous research supporting the notion that binary distinctions between gender roles may not be sufficient to explain differences in functional outcomes post-TBI.^32^ It should be noted that the presented prognostic factors for deterioration should not be mixed with prognostic factors for early probability of survival, given that this study only included long-term survivors.

Quality of life among patients in this TBI cohort (median, 0.88 [0.66–1.00]) was similar to European and TBI populations, varying around 0.80–0.95 (mean).^37–41^ The only past study assessing the relationship between GOS and EQ-5D-5L in TBI survivors found a median index score of 0.93 upon hospital discharge. The high index scores of TBI patients is supported by recent research suggesting TBI patients to have higher perceived quality of life (EQ-5D-3L) compared to other trauma patients 2 years post-trauma.^42^ In this study, higher GOS scores coincided with higher index scores, supporting a positive correlation between GOS and index scores proposed previously.^27^ However, GOSE 5 did not increase similarly to the other GOSE categories, suggesting a higher perceived quality of life in the severe compared to the lower end of the moderate disability group.^43,44^

We found significantly higher index scores for patients whose functional outcomes remained the same/improved over time. Most patients (93%) with stable/improved functional outcomes had moderate disability or good recovery (GOS 4–5), suggesting functional disability to importantly determine life quality. The correlation between index scores and VAS was weaker than found previously. However, the previously found median VAS of 98^27^ is higher than average VAS scores observed in general populations.^37–41^ Self-rated (VAS) could be higher upon hospital discharge compared to at 15 years post-TBI after having been burdened by non-TBI problems.

### Limitations

First, because of the longitudinal design of the study, many patients initially recruited were either dead or lost to follow up.^33^ Due to the small sample size, analyses to detect variables contributing independently to long-term outcome deterioration could not be performed. However, the sample size of the current study is similar to that of ∼100 of other 10- to 15-year post-TBI longitudinal studies.^12,18,20^

Second, only 4 patients with early poor functional outcome were alive and replied to the questionnaires at 15 years. Thus, functional deterioration was the only possible trajectory of recovery for the majority of patients.

Third, outcome measures (GOS, GOSE, and EQ-5D-5L) are, to some extent, influenced by assessment technique of the rater or the patient.^45,46^ In the future, data should be obtained in the form of either GOS or GOSE instead of as different measures. Collecting data in letter format and over the phone could have increased the variability between ratings, but simultaneously increased sample size.

Fourth, this study does not assess the effect of socioeconomical parameters, lifestyle factors (such as physical activity and alcohol consumption), comorbidities, and changes in comorbidities, on long-term neurological outcome and neurological deterioration in TBI survivors, which could have influenced outcome measures independently of the TBI.^47^

## Conclusion

Nearly all long-term TBI survivors had an initial favorable functional outcome, indicating that long-term survival in those with an unfavorable neurological outcome is rare. Functional outcome deteriorated in almost half of patients during long-term follow-up. Higher age and diffuse brain injury were associated with neurological deterioration. Quality of life was strongly linked to functional outcome.

## Supplementary Material

Supplemental data

Supplemental data

Supplemental data

Supplemental data

Supplemental data

Supplemental data

Supplemental data

Supplemental data

## Supplementary Material

STROBE

## Abbreviations Used

CIs: confidence intervals
CT: computed tomography
EQ-5D-5L: EuroQol Five Dimensions Five Levels
GCS: Glasgow Coma Scale
GOS: Glasgow Outcome Scale
GOSE: Glasgow Outcome Scale-Extended
ICU: intensive care unit
IQR: interquartile range
TBI: traumatic brain injury
VAS: Visual Analog Scale
VIF: variance inflation factor
