# Supplementary material for: Long-Term Functional Outcome and Quality of Life in Long-Term Traumatic Brain Injury Survivors
Source: Neurotrauma Rep. 2023 Nov 22;4(1):813–22. doi: 10.1089/neur.2023.0064 (PMC10698799; doi:10.1089/neur.2023.0064)
Supplement: Supplemental data [file Suppl_Data.docx]

STROBE Statement—checklist of items that should be included in reports of observational studies

|  | Item No. | Recommendation | Page  No. | Relevant text from manuscript |
| --- | --- | --- | --- | --- |
| **Title and abstract** | 1 | (*a*) Indicate the study’s design with a commonly used term in the title or the abstract |  | “Prospective, longitudinal study” |
|  |  | (*b*) Provide in the abstract an informative and balanced summary of what was done and what was found |  | “Early functional outcome…to functional outcome.” |
| Introduction | | | |  |
| Background/rationale | 2 | Explain the scientific background and rationale for the investigation being reported |  | “Traumatic brain injury…and GOS scores ^27^.” |
| Objectives | 3 | State specific objectives, including any prespecified hypotheses |  | “This study aimed…higher life quality ^27^.” |
| Methods | | | |  |
| Study design | 4 | Present key elements of study design early in the paper |  | “This prospective longitudinal…the Finnish population”. |
| Setting | 5 | Describe the setting, locations, and relevant dates, including periods of recruitment, exposure, follow-up, and data collection |  | “This prospective, longitudinal…during 10.4.2000–21.11.2002.”  “We assessed early…(GOSE, January 2018-July 2018).”  “The GOS questionnaires…made via telephone.”  “We measured quality of life using the EQ-5D-5L at 15 years post-injury.”  “The EQ-5D-5L questionnaire…made via telephone.” |
| Participants | 6 | (*a*) *Cohort study*—Give the eligibility criteria, and the sources and methods of selection of participants. Describe methods of follow-up |  |  |
| Variables | 7 | Clearly define all outcomes, exposures, predictors, potential confounders, and effect modifiers. Give diagnostic criteria, if applicable |  | “Age was considered…into one class (V).” |
| Data sources/ measurement | 8* | For each variable of interest, give sources of data and details of methods of assessment (measurement). Describe comparability of assessment methods if there is more than one group |  | “Age was considered…neurosurgeon and recorded.”  “The GOS questionnaires…early post-injury recovery ^10^.”  “The EQ-5D-5L questionnaire…contact via telephone.” |
| Bias | 9 | Describe any efforts to address potential sources of bias |  | **eTable 1** |
| Study size | 10 | Explain how the study size was arrived at |  | “This prospective, longitudinal…were not excluded.” |

| Quantitative variables | 11 | Explain how quantitative variables were handled in the analyses. If applicable, describe which groupings were chosen and why |  | “For early functional…5–8 as favorable.”  “The EQ-5D-5L was…the Danish value set ^29^.” |
| --- | --- | --- | --- | --- |
| Statistical methods | 12 | (*a*) Describe all statistical methods, including those used to control for confounding |  | “We presented categorical…index score analyses.” |
|  |  | (*b*) Describe any methods used to examine subgroups and interactions |  | “We created two… (early GOS 4–5 to late GOSE 1–4).”  “We used Box-Tidwell…functional outcomes deteriorated.” |
|  |  | (*c*) Explain how missing data were addressed |  | “Patients with one missing value for any predictor were excluded from the regression analyses.”  “Patients with one missing value for any EQ-5D-5L dimension were excluded from the index score analyses.” |
|  |  | (*d*) *Cohort study*—If applicable, explain how loss to follow-up was addressed  *Case-control study*—If applicable, explain how matching of cases and controls was addressed  *Cross-sectional study*—If applicable, describe analytical methods taking account of sampling strategy |  |  |
|  |  | (*e*) Describe any sensitivity analyses |  |  |
| Results | | | | |
| Participants | 13* | (a) Report numbers of individuals at each stage of study—eg numbers potentially eligible, examined for eligibility, confirmed eligible, included in the study, completing follow-up, and analysed |  | “Of the 698…to follow-up questionnaires.”  **Figure 1** |
|  |  | (b) Give reasons for non-participation at each stage |  | **Figure 1:** “Excluded due to…” |
|  |  | (c) Consider use of a flow diagram |  | **Figure 1** |
| Descriptive data | 14* | (a) Give characteristics of study participants (eg demographic, clinical, social) and information on exposures and potential confounders |  | “Differences in baseline…improved functional outcome (**Table 2**).” |
|  |  | (b) Indicate number of participants with missing data for each variable of interest |  | “Of the 118 patients…(5 due to missing GCS, 3 due to missing pupillary reactivity).”  “Of the 118 patients…not be derived.” |
|  |  | (c) *Cohort study*—Summarise follow-up time (eg, average and total amount) |  |  |
| Outcome data | 15* | *Cohort study*—Report numbers of outcome events or summary measures over time |  |  |
|  |  | *Case-control study—*Report numbers in each exposure category, or summary measures of exposure |  |  |
|  |  | *Cross-sectional study—*Report numbers of outcome events or summary measures |  |  |
| Main results | 16 | (*a*) Give unadjusted estimates and, if applicable, confounder-adjusted estimates and their precision (eg, 95% confidence interval). Make clear which confounders were adjusted for and why they were included |  | **Table 3:** “OR, 95% CI” |
|  |  | (*b*) Report category boundaries when continuous variables were categorized |  | **Table 3**: “**Sex**, female as the reference category  **Pupil responsiveness,** unresponsive (unilaterally + bilaterally) as the reference category  **Marshall CT,** II used as the reference category” |
|  |  | (*c*) If relevant, consider translating estimates of relative risk into absolute risk for a meaningful time period |  |  |

Con

| Other analyses | 17 | Report other analyses done—eg analyses of subgroups and interactions, and sensitivity analyses |  | “The Box-Tidwell…or little multicollinearity.”  “A Mann-Whitney U…(n=53) (mean rank=36.59), (p<0.001, **eFigures 2–4**).” |
| --- | --- | --- | --- | --- |
| Discussion | | | | |
| Key results | 18 | Summarise key results with reference to study objectives |  | “This prospective, longitudinal…and late measurements.” |
| Limitations | 19 | Discuss limitations of the study, taking into account sources of potential bias or imprecision. Discuss both direction and magnitude of any potential bias |  | “First, because of…independently of TBI ^44^.” |
| Interpretation | 20 | Give a cautious overall interpretation of results considering objectives, limitations, multiplicity of analyses, results from similar studies, and other relevant evidence |  | “This study presents…by non-TBI problems.” |
| Generalisability | 21 | Discuss the generalisability (external validity) of the study results |  | “Our sample consisted…than found previously ^12,18–21^.”  “Marshall CT IV was not associated with neurological deterioration, probably due to the high mortality among these injuries.” |
| Other information | |  | | |
| Funding | 22 | Give the source of funding and the role of the funders for the present study and, if applicable, for the original study on which the present article is based |  | “The study was funded by a research grant from Helsinki University Hospital.” |

*Give information separately for cases and controls in case-control studies and, if applicable, for exposed and unexposed groups in cohort and cross-sectional studies.

**Note:** An Explanation and Elaboration article discusses each checklist item and gives methodological background and published examples of transparent reporting. The STROBE checklist is best used in conjunction with this article (freely available on the Web sites of PLoS Medicine at http://www.plosmedicine.org/, Annals of Internal Medicine at http://www.annals.org/, and Epidemiology at http://www.epidem.com/). Information on the STROBE Initiative is available at www.strobe-statement.org.
