# Supplementary material for: Long-Term Functional Outcome and Quality of Life in Long-Term Traumatic Brain Injury Survivors
Source: Neurotrauma Rep. 2023 Nov 22;4(1):813–22. doi: 10.1089/neur.2023.0064 (PMC10698799; doi:10.1089/neur.2023.0064)
Supplement: Supplemental data [file Suppl_TableS1.docx]

**eTable 1:** Differences in characteristics between patients who replied and did not reply to functional outcome and quality of life surveys.

| **Variable** | **Patients replying to functional outcome and quality of life surveys at 15 years**  **(N=118)** | **Patients not replying to functional outcome surveys**  **(N=62)** |
| --- | --- | --- |
| **Age of admission**, median (IQR)^[[1]](#footnote-1)^ | 34 (19-45) | 41 (25-51) |
| **Gender** |  |  |
| Male | 88 (75%) | 52 (84%) |
| Female | 30 (25%) | 10 (16%) |
| **GCS^[[2]](#footnote-2)^ score** |  |  |
| 3-8 | 50 (42%) | 25 (40%) |
| 9-12 | 29 (25%) | 16 (26%) |
| 13-15 | 34 (29%) | 16 (26%) |
| NA^[[3]](#footnote-3)^ | 5 (4%) | 5 (8%) |
| **Pupil responsiveness** |  |  |
| Bilaterally unresponsive | 11 (9%) | 4 (6%) |
| Unilaterally unresponsive | 11(9%) | 7 (11%) |
| Responsive | 93 (79%) | 50 (81%) |
| NA | 3 (3%) | 1 (2%) |
| **Marshall CT** |  |  |
| I | 0 (0%) | 0 (0%) |
| II | 70 (59%) | 30 (48%) |
| III | 15 (13%) | 6 (10%) |
| IV | 7 (6%) | 4 (6%) |
| V | 26 (22%) | 22 (36%) |
| **Cause of injury** |  |  |
| Fall from ground level | 33 (28%) | 28 (45%) |
| Fall from height | 13 (11%) | 3 (4%) |
| Traffic accident | 40 (34%) | 9 (15%) |
| Interpersonal violence | 10 (8%) | 5 (8%) |
| Other | 14 (12%) | 8 (13%) |
| Unknown | 8 (7%) | 9 (15%) |
| *All percentages rounded to the nearest whole number.* | | |

1. *IQR=Interquartile Range* [↑](#footnote-ref-1)
2. *GCS=Glasgow Coma Scale* [↑](#footnote-ref-2)
3. *NA=Not Accessible* [↑](#footnote-ref-3)
