# Supplementary material for: Long-Term Functional Outcome and Quality of Life in Long-Term Traumatic Brain Injury Survivors
Source: Neurotrauma Rep. 2023 Nov 22;4(1):813–22. doi: 10.1089/neur.2023.0064 (PMC10698799; doi:10.1089/neur.2023.0064)
Supplement: Supplemental data [file Suppl_TableS2.docx]

**eTable 2:** Differences in characteristics between patients who replied to functional outcome and quality of life surveys and patients who died before the 15-year follow-up

| **Variable** | **Patients replying to functional outcome and quality of life surveys at 15 years**  **(N=118)** | **Patients who died before the 15-year follow-up**  **(N=162)** |
| --- | --- | --- |
| **Age of admission**, median (IQR)^[[1]](#footnote-1)^ | 34 (19-45) | 55 (45-67) |
| **Gender** |  |  |
| Male | 88 (75%) | 119 (73%) |
| Female | 30 (25%) | 43 (27%) |
| **GCS score^[[2]](#footnote-2)^** |  |  |
| 3-8 | 50 (42%) | 74 (46%) |
| 9-12 | 29 (25%) | 36 (22%) |
| 13-15 | 34 (29%) | 38 (23%) |
| NA^[[3]](#footnote-3)^ | 5 (4%) | 14 (9%) |
| **Pupil responsiveness** |  |  |
| Bilaterally unresponsive | 11 (9%) | 28 (18%) |
| Unilaterally unresponsive | 11(9%) | 15 (9%) |
| Responsive | 93 (79%) | 117 (72%) |
| NA | 3 (3%) | 2 (1%) |
| **Marshall CT*** |  |  |
| I | 0 (0%) |  |
| II | 70 (59%) |  |
| III | 15 (13%) |  |
| IV | 7 (6%) |  |
| V | 26 (22%) |  |
| **Cause of injury** |  |  |
| Fall from ground level | 33 (28%) | 104 (64%) |
| Fall from height | 13 (11%) | 8 (5%) |
| Traffic accident | 40 (34%) | 14 (9%) |
| Interpersonal violence | 10 (8%) | 4 (2%) |
| Other | 14 (12%) | 8 (5%) |
| Unknown | 8 (7%) | 24 (15%) |
| *All percentages rounded to the nearest whole number.*  **Marshall CT not assessed for non-survivors* | | |

1. *IQR=Interquartile Range* [↑](#footnote-ref-1)
2. *GCS=Glasgow Coma Scale* [↑](#footnote-ref-2)
3. *NA=Not Accessible* [↑](#footnote-ref-3)
