# Supplementary material for: Long-Term Functional Outcome and Quality of Life in Long-Term Traumatic Brain Injury Survivors
Source: Neurotrauma Rep. 2023 Nov 22;4(1):813–22. doi: 10.1089/neur.2023.0064 (PMC10698799; doi:10.1089/neur.2023.0064)

**eFigure 5**: Kaplan-Meier curve demonstrating cumulative survival for all patients who agreed to participate in the study (n=342). The sharpest drop off for survival occurred during the first month, during which 30 patients died. Following the first month, the drop in survival was more gradual than during the first month. 180 patients (53%) were still alive at the time of the 15-year follow-up.


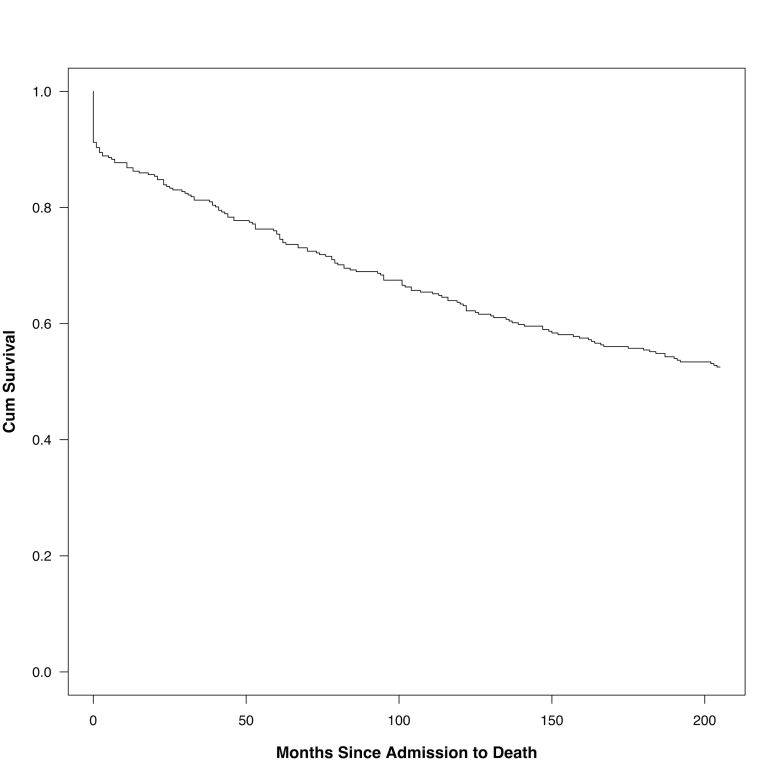

Supplement: Supplemental data [file Suppl_FigureS5.docx]
