# Supplementary material for: Long-Term Functional Outcome and Quality of Life in Long-Term Traumatic Brain Injury Survivors
Source: Neurotrauma Rep. 2023 Nov 22;4(1):813–22. doi: 10.1089/neur.2023.0064 (PMC10698799; doi:10.1089/neur.2023.0064)
Supplement: Supplemental data [file Suppl_FigureS1.docx]

**eFigure 1**: Scatter plot and linear regression of EQ-5D-5L index score and EQ-5D-5L Visual Analogue Scale (VAS). Fifty six percent of the variance in index score was explained by VAS, indicating that there is a moderate positive correlation between index scores and VAS.
