# Supplementary material for: Long-Term Functional Outcome and Quality of Life in Long-Term Traumatic Brain Injury Survivors
Source: Neurotrauma Rep. 2023 Nov 22;4(1):813–22. doi: 10.1089/neur.2023.0064 (PMC10698799; doi:10.1089/neur.2023.0064)
Supplement: Supplemental data [file Suppl_TableS4.docx]

**eTable 4:** Multiple linear regression to predict EQ-5D-5L index scores

| **Variable** | **Standardized coefficient beta (**OR, 95% CI) | **Tolerance** | **VIF^[[1]](#footnote-1)^** |
| --- | --- | --- | --- |
| **Age of admission** | -0.15 (-0.01-0.00) | 0.75 | 1.34 |
| **Sex**, female as the reference category | -0.04 (-0.14-0.09) | 0.97 | 1.03 |
| **GCS^[[2]](#footnote-2)^ score** | 0.02 (-0.01-0.01) | 0.74 | 1.35 |
| **Pupil responsiveness,** as a continuous variable | -0.02 (-0.15-0.13) | 0.77 | 1.30 |
| **Marshall CT,** as a continuous variable | -0.10 (-0.07-0.03) | 0.72 | 1.40 |

1. *Variance Inflation Factor=VIF* [↑](#footnote-ref-1)
2. *GCS=Glasgow Coma Scale* [↑](#footnote-ref-2)
