# Supplementary material for: Long-Term Functional Outcome and Quality of Life in Long-Term Traumatic Brain Injury Survivors
Source: Neurotrauma Rep. 2023 Nov 22;4(1):813–22. doi: 10.1089/neur.2023.0064 (PMC10698799; doi:10.1089/neur.2023.0064)
Supplement: Supplemental data [file Suppl_FigureS2-S4.zip › Suppl_FigureS2-4.docx]

**eFigure 2**: Histogram of EQ-5D-5L index score frequency for all patients. *Normal curve shown in figure.* The mean (SD) index score for all patients with index scores (n=116) was 0.78 (0.26). Fifty percent of values fell between 0.88-1.00 (n=62, 53%). A Mann-Whitney U test was used to assess whether index scores of patients whose outcomes remained stable or improved differed significantly from those whose index scores deteriorated between early and late functional outcome measurements.

**eFigure 3**: Histogram of EQ-5D-5L index score frequency for patients with stable or improved functional outcome between early and late measurement**.** *Normal curve shown in figure.* The mean (SD) index score for patients (n=63) whose functional outcome was stable or improved was 0.90 (1.81). Fifty percent of patients had an index score of 1.00 (n=33, 52%). As the shape of the distributions of this histogram and **eFigure 4** are not similar and Levene’s test p<0.001, the Mann-Whitney U test was used to compare the mean ranks between the two groups.

**eFigure 4**: Histogram of EQ-5D-5L index score frequency for patients whose functional outcomes deteriorated between early and late measurement. *Normal curve shown in figure.* The mean (SD) index score for patients (n=53) whose functional outcome deteriorated was 0.64 (0.27). Fifty percent of values fell between 0.69-1.00 (n=27, 51%). As the shape of the distributions of this histogram and **eFigure 3** are not similar and Levene’s test p<0.001, the Mann-Whitney U test was used to compare the mean ranks between the two groups.
