# Supplementary figures and images for: Long-Term Functional Outcome and Quality of Life in Long-Term Traumatic Brain Injury Survivors
Source: Neurotrauma Rep. 2023 Nov 22;4(1):813–22. doi: 10.1089/neur.2023.0064 (PMC10698799; doi:10.1089/neur.2023.0064)

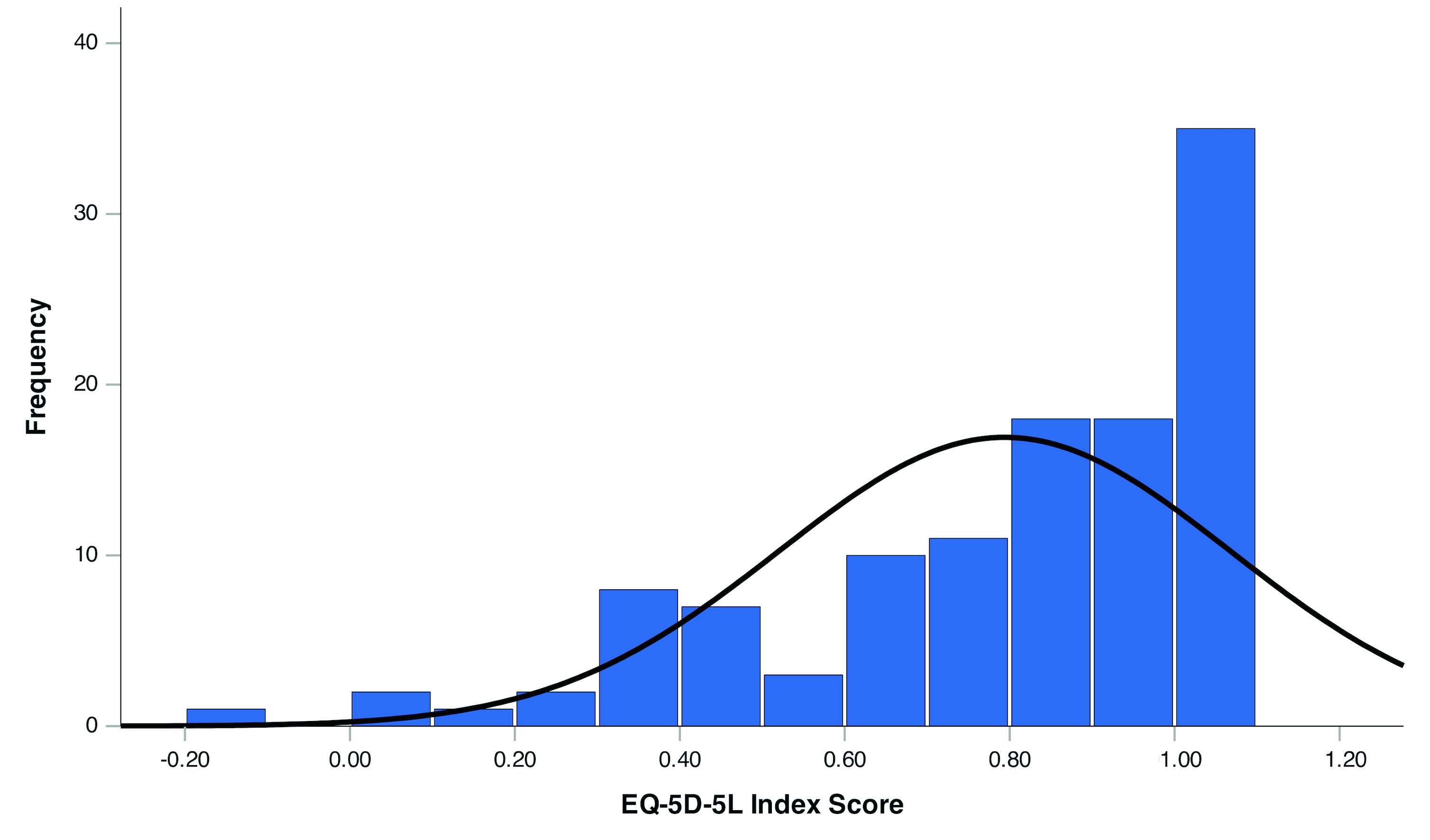

Supplement: Supplemental data [file Suppl_FigureS2-S4.zip › TaalasSupplementaryFig2.tif]

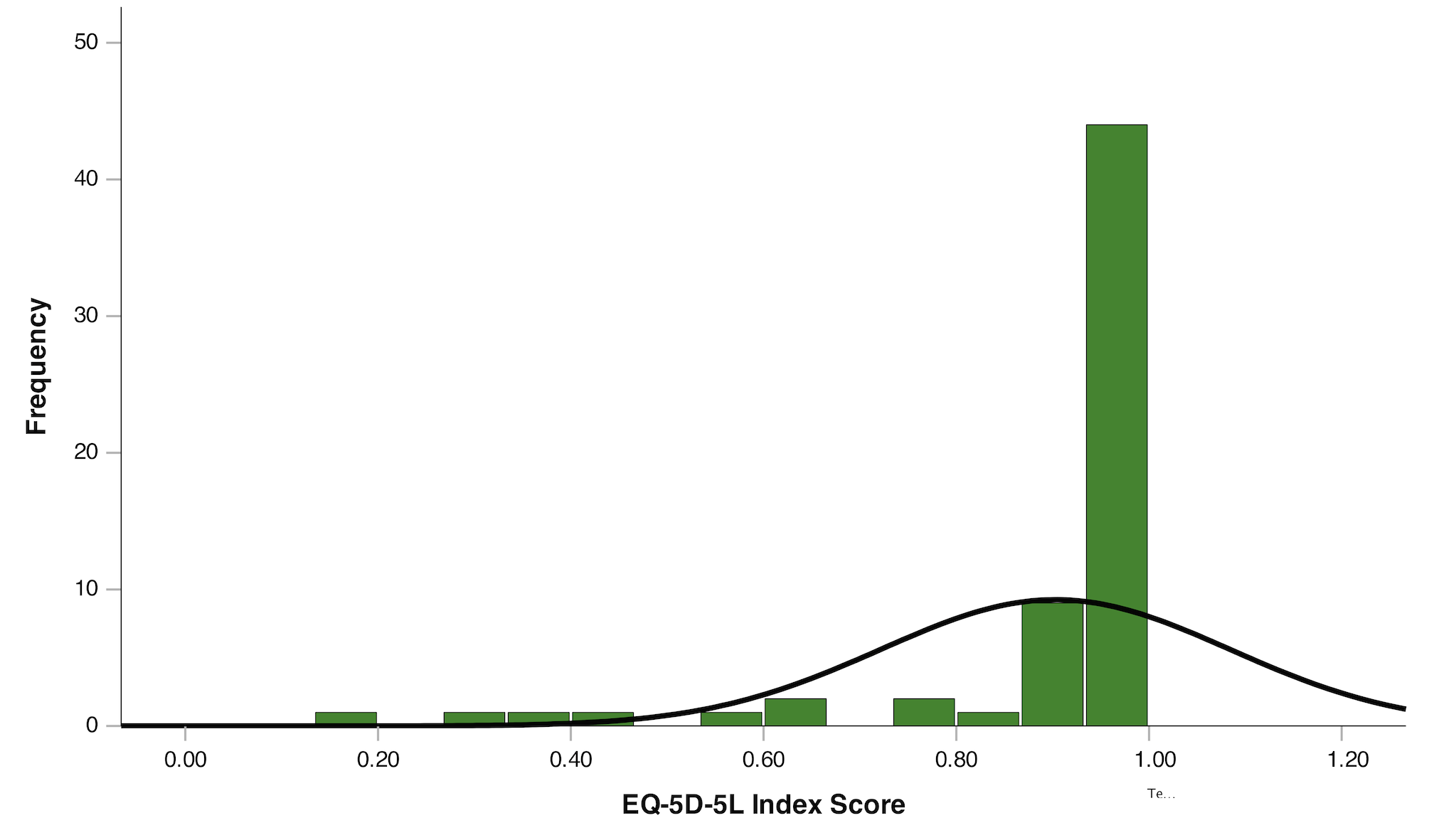

Supplement: Supplemental data [file Suppl_FigureS2-S4.zip › TaalasSupplementaryFig3.tif]

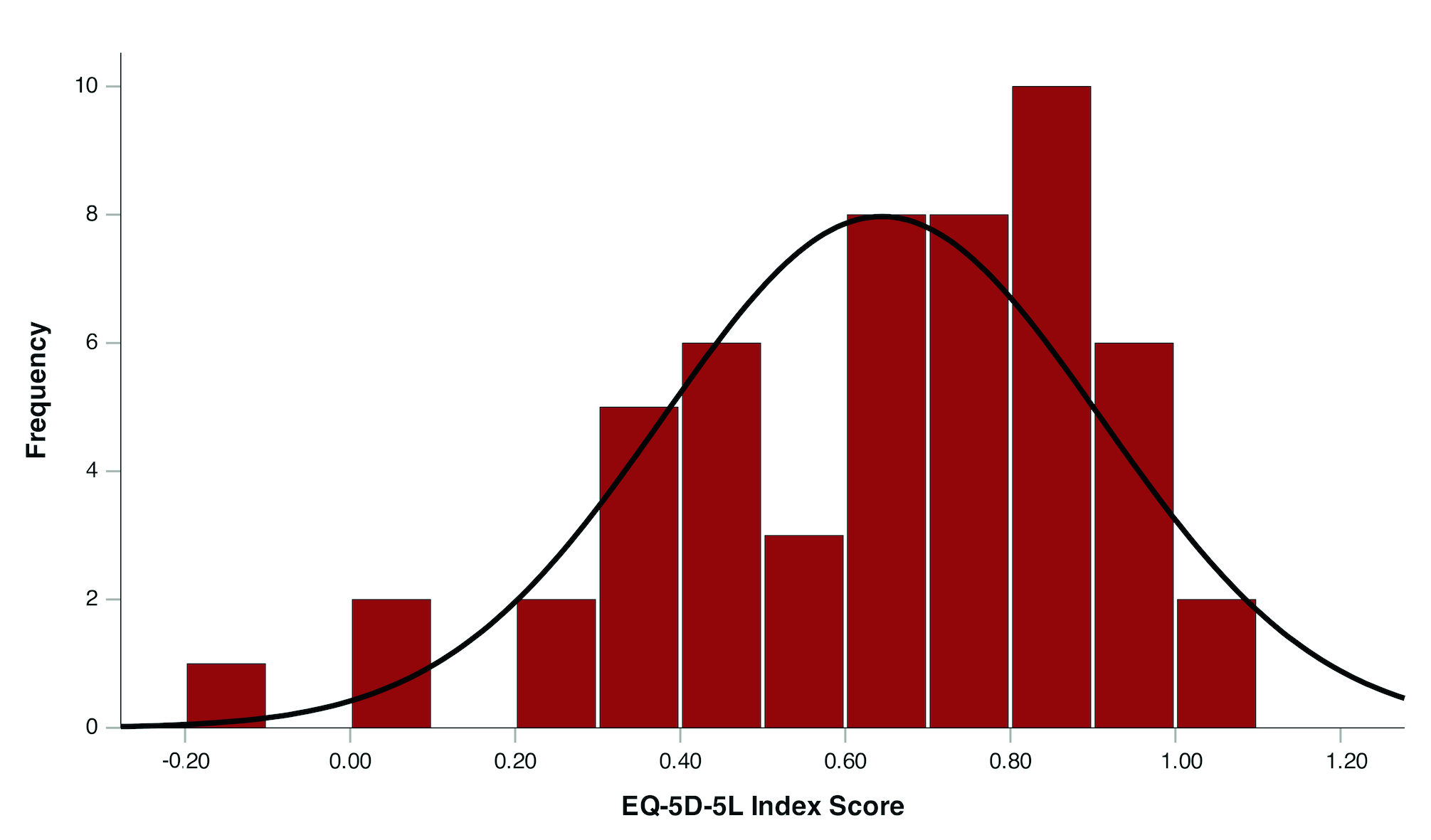

Supplement: Supplemental data [file Suppl_FigureS2-S4.zip › TaalasSupplementaryFig4.tif]
